# Epidemiology of *tunga penetrans* infestation in selected areas in Kiharu constituency, Murang’a County, Kenya

**DOI:** 10.1186/s40794-015-0015-4

**Published:** 2015-12-05

**Authors:** Jamleck N. Mwangi, Hastings S. Ozwara, Michael M. Gicheru

**Affiliations:** 1 Department of Zoological sciences, Kenyatta University, P. O. Box 43844-00100, Nairobi, Kenya; 2 Department of Tropical and Infectious Diseases, Institute of Primate Research, National Museums of Kenya, P. O. Box 24481, Karen, 00502 Nairobi Kenya

**Keywords:** Epidemiology, Tungiasis, Factors associated with tungiasis

## Abstract

**Background:**

Tungiasis is a parasitic skin disease brought about by female *Tunga penetrans* when they burrow into the skin of their hosts. It is a disease that has largely been ignored. Epidemiology of tungiasis has not been widely studied in Kenya which could negatively affect effective intervention strategies. This study therefore sought to investigate epidemiology of tungiasis in selected areas in Kiharu constituency, Murang’a County in Kenya.

**Methods:**

The study population comprised of public primary school pupils, the most vulnerable age group (*n* = 508) in Gaturi, Kimathi, Kahuhia and Mugoiri in Kiharu constituency. Public primary school pupils in the study area were randomly sampled. Through questionnaires and observations, data was collected.

**Results:**

The overall prevalence of tungiasis in pupils in the study area was 19.1 %. In multinomial logistic regression analysis some factors were identified to be associated with tungiasis such as lack of regular use of closed foot ware (Adjusted odds ratio = 10.45; 95 % Confidence Interval; 1.49–73.23), living in earthen mud walled houses (aOR = 13.78; 95 % CI = 3.127–60.69), sharing living quarters with domestic animals (aOR = 3.1; 95 % CI = 0.003–.046) and learning in classrooms with dusty floors (aOR = 14.657; 95 % CI = 2.262–94.95). Treatment of tungiasis was found to be mainly through mechanical removal of embedded *T. penetrans*.

**Conclusion:**

This study shows that tungiasis in the selected study areas of Kiharu constituency is a disease of significant health concern. Factors associated with tungiasis were identified that should be the focus of sustainable and effective control measures.

## Background

Tungiasis is a parasitic skin disease caused by female *T. penetrans* [1]. Signs associated with the disease include severe local itching, pain and sensation of a foreign body in the skin [2]. Most lesions occur on the nail rim, periungual area of the toes, the heels, the soles and other parts of the body such as hands, elbows, neck, buttocks and the genital region [1, 3–5]. Severe lesions do occur and are usually located in clusters [6]. Moreover, heavy infestation with hundred of embedded *T. penetrans* often occur in severe tungiasis [6]. Diagnosis involves identification of the parasite especially through mechanical removal using a sharp pointed object such as a needle. Furthermore, patient’s travelling history is also quite important.

Tungiasis is endemic in many countries in Latin America, the Caribbean and sub-Sahara Africa [2]. In Kenya, it is a neglected serious health problem to the extent that epidemiological data is scarce [3]. It has led to severe morbidity to its victims especially in economically challenged rural communities [7]. More over tungiasis is incapacitating especially due to severe physical disability emanating from its pathological effects especially on limbs. This often contributes to the characteristic high poverty levels to it victims. In Nigeria, a third of people with tungiasis have difficulties in walking [8]. Tungiasis high transmission rate is due to some factors such as poor housing conditions, social neglect and inadequate health care [2]. In Erekit, a rural community in Lagos, Nigeria, earthen houses, presence of loitering pigs, resting in a common place near the house, and lack of regular use of closed foot ware are important factors for tungiasis [8]. In a fishing rural community North East Brazil, intensity of infestation in infested pets was found to positively correlate to intensity of infestation among the members of the community [9].

As of 2009, estimated 1.6 million Kenyans were suffering from tungiasis and 10 million others were at risk [10]. In 2010, the prevalence of tungiasis in Murang’a south, an endemic area, was suggested to be 57 % in children of 5–12 years [11]. Unhygienic conditions have been identified as the major causes of tungiasis in Kenya [12]. In addition, soil factors such as organic matter content, moisture, pH, color and texture have been suggested to influence prevalence of tungiasis by up to 33 % [13]. Soil factors were also found to influence *T. penetrans* population by up to 39.7 % [13]. Though such information is already available, more work on epidemiology especially in vulnerable age groups such as children and the aged in other endemic regions in Kenya need to be pursued. In fact, the prevalence rates in different Counties in Kenya are not very clear [3]. Murang’a is one of the Counties suggested to have high prevalence of tungiasis [3].

## Methods

### Study population, study area and study design

This study was carried out in 2012 and 2013 when the public primary schools were in session, during dry weather seasons. This is because rainy seasons made some places inaccessible due to poor road network. Before the onset of the study, information meetings were held with teachers, parents, pupils and community members. Areas recruited for this study are Gaturi, Kimathi, Kahuhia and Mugoiri in Kiharu constituency that has a total of 21 public primary schools. Their accessibility was important while reports of tungiasis in primary schools from local health officers were useful during survey. All 21 schools (Gaturi 5, Mugoiri 8, Kimathi 4 and Kahuhia 4 schools) with a total population of 3500 pupils were approached and sampled. Five hundred and eight pupils participated in the study. Simple random sampling was used to recruit pupils for the study using a random number table. Observations and pilot tested questionnaires were used to collect data at school. Pupil’s homes were visited to take home data.

Diagnosis of tungiasis was done by a clinical officer. All participants were thoroughly examined for the presence of embedded *T. penetrans*. Clinical examination was carefully performed by inspecting feet, legs, hands and arms for lesions in various stages of Fortaleza classification. To uphold individual privacy, other parts of the body not normally exposed were not examined [14]. For the purpose of clinical examination the following was considered diagnostic for tungiasis: an itchy spot that is either red to white-brown in color, a lesion that is circular with a black dot at the center, at times with strings of white tiny eggs oozing out. Other diagnostic findings were a crust that is black with necrotic tissues, including totally or partially removed fleas, hence a characteristic sore skin. Localization and number of lesions were recorded and categorized as mild (<5 lesions), moderate (6 to 30 lesions) and heavy infestation (>30 lesions) [15].

### Inclusion criteria and exclusion criteria

Pupils sampled in the study population, both infested and non-infested by *T. penetrans* were included in the study. However pupils who reserved their consent were excluded.

### Ethical approval and consent

Written research ethical clearance was obtained from Murang’a general hospital ethical committee and Institute of Primate Research (IPR). Written permits to visit schools were obtained from Education Officers in the study area. Before the study, the objectives and the study protocol were explained to the teachers and pupils in meetings who then approved the study. Written consent by the head teachers was given. Each study subject also gave written consent at individual level.

### Statistical analysis

IBM SPSS version 20 was used to analyze the data. Binary logistic regression analysis of the variables was first performed to determine variables for analysis in a multinomial logistic analysis. Variables whose p value was less than 0.05 in the binary analysis were adjusted for confounding in a multinomial logistic analysis. Their adjusted odds ratios were calculated to determine their independent association with tungiasis. Colinearity of the variables was assessed using regression diagnostics. Variables analyzed using multinomial logistic regression showed no collinearity. Statistical significance was considered when value of *p* ≤ 0.05.

## Results

**Fig. 1:**
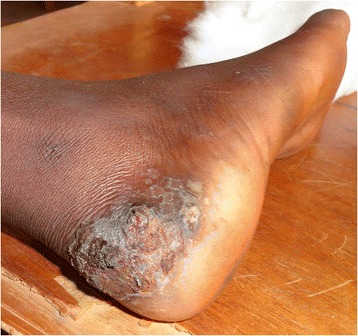
Lesions of *T. penetrans* on the right heel of a grade 6 boy

**Fig. 2:**
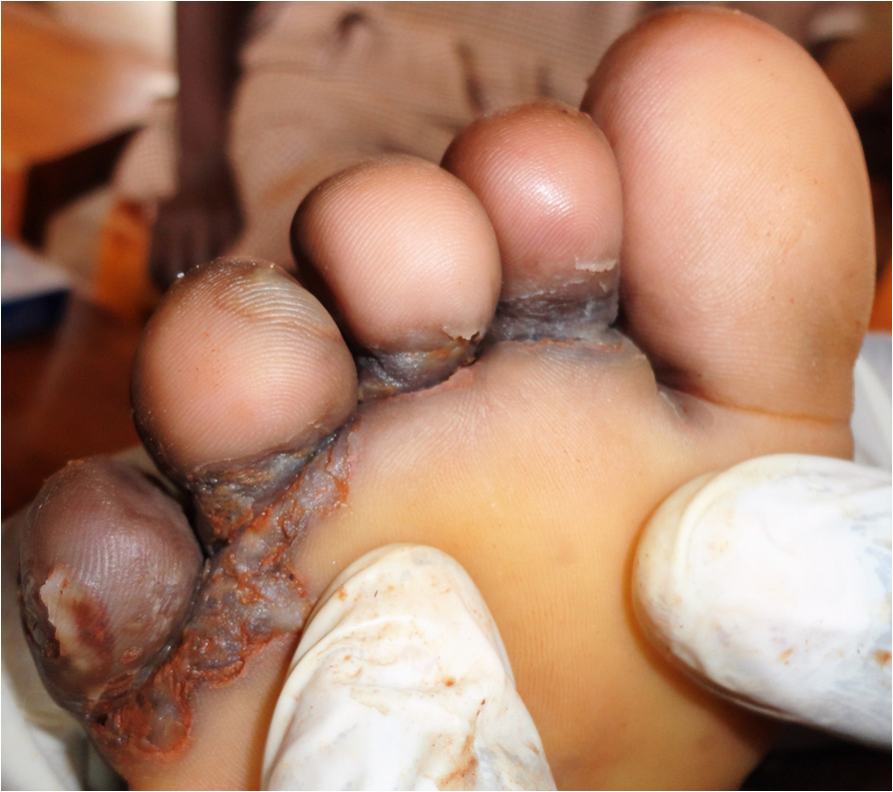
Lesions of *T. penetrans* in the peringual region of the left foot of a grade 4 girl

**Fig. 3:**
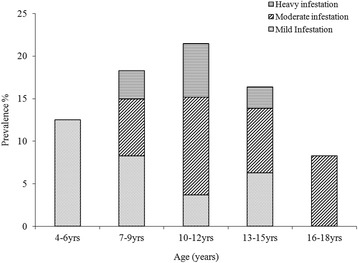
Prevalence of *T. penetrans* infestation stratified by different age brackets and severity of tungiasis

Data was collected from 508 pupils that participated in the study. Boys were 259 (50.98 %) and girls were 249 (49.01 %). Tunga penetrans lesions were mainly found on feet (Figs. 1 and 2). Pupils infested with *T. penetrans* were 97, a prevalence of 19.1 %. The prevalence of tungiasis in pupils in Mugoiri was 23.3 % (45/193), 21.6 % (21/97) in Kahuhia, 17.5 % (17/97) in Kimathi and 11.5 % (14/121) in Gaturi. A total of 52 boys were infested, a prevalence of 20.1 % while 45 girls were infested which was a prevalence of 18.1 %. Pupils with mild infestation were 26 (5.1 %), moderately infested were 48 (9.4 %) and those heavily infested were 23 (4.5 %). The intensities of infestation related to pupil’s age are shown in Fig. 3. The prevalence of tungiasis in pupils in relation to their age brackets was observed to have a unique trend that assumed a sigmoid curve. This is because pupils of 4–6 years had a prevalence of 12.5 %, while those of 7–9 years had a prevalence of 18.3 %. The peak was in children of 10–12 years whose prevalence was 21.5 %. This prevalence then decreased so that children between 13–15 years had a prevalence of 16.5 % and those of 16–18 years had a prevalence of 8.3 %.

The odds ratios of factors identified to influence tungiasis in a binary logistic analysis are shown in Table 1. In a multinomial logistic regression (Table 2), factors independently associated with tungiasis were identified as; type of housing material (adjusted odds ratio = 13.78; 95 % Confidence Interval = 3.127–60.69), Sharing living quarters with domestic animals (aOR = 3.1; 95 % CI = 0.003–0.046), lack of closed foot wears (aOR = 10.45; 95 % CI = 1.49–73.23) and status of classroom floors (aOR = 14.657; 95 % CI = 2.262–94.95).

**Table 1:** Binary logistic regression analysis of factors associated with tungiasis

Factor	No. examined (n)	Infested (%)	Odds ratio (95 % CI)	*p* value
Gender				
Girls	249	45 (18.07)	Reference	
Boys	259	52 (20.07)	0.622 (0.158–2.45)	0.498
Housing wall material				
Wood	179	4 (2.23)	0.009 (0.01–0.077)	0.000
Iron sheet	188	17 (9.04)	0.063 (0.013–0.303)	0.001
Mud/mud bricks	141	76 (53.9)	Reference	
Sharing living quarters with domestic animals				
Share	96	80 (83.3)	123.63 (22.68–673.95)	0.000
Do not share	412	17 (4.1)	Reference	
Status of classroom floor				
Cemented and clean	399	11 (2.7)	0.002 (0.000–0.015)	0.000
Not cemented	26	14 (53.8)	0.062 (0.008–0.460)	0.007
Cemented and dusty	83	72 (86.7)	Reference	
Footwear				
With shoes	77	6 (7.79)	0.081 (0.010–0.661)	0.019
Without shoes	431	91 (21.1)	Reference	
Knowledge				
(a) Probable cause of tungiasis				
From soil	158	27 (17.1)	0.640 (0.072–5.713)	0.690
From Domestic animals	269	52 (19.3)	1.095 (0.153–7.854)	0.928
From infested people	81	18 (22.2)	Reference	
(b) Treatment employed				
Do not treat	14	6 (42.8)	0.142 (0.001–18.177)	0.430
Using herbs	48	7 (14.58)	0.147 (0.004–5.810)	0.307
Removing them	415	76 (18.31)	0.448 (0.023–8.781)	0.597
Removing and use of herbs	31	8 (25.8)	Reference

**Table 2:** Multinomial logistic regression analysis of factors associated with tungiasis

Factor	Adjusted OR (95 % CI)	*P* value
Conditions of classroom floor	14.656 (2.262–94.951)	0.005
Housing materials	13.775 (3.127–60.685)	0.001
Sharing living quarters with domestic animals	3.1 (0.003–0.046)	0.000
Use of closed foot ware	10.448 (1.491–73.228)	0.018

## Discussions

Most pupils preferred treatment of tungiasis by immediate mechanical removal of embedded fleas using sharp pointed objects. This destroys the flea before it lays eggs, break the life cycle and also prevent secondary infections. However there is danger of sharing contaminated sharp objects among pupils that can spread blood related diseases such as HIV and hepatitis. In view of this observation, exploring other effective ways of treating tungiasis without actual mechanical removal, such as washing with disinfectant, topographic application of anti-parasitic agents, use of anti-inflammatory creams and use of repellants should be preferred.

Domestic animals in the home such as cats, dogs, chicken and goats is an important factor associated with tungiasis, perhaps because they harbor the *T. penetrans* fleas. Moreover sharing house with such animals aggravates tungiasis. Similarly, the importance of such domestic animals has been described in a study done in traditional fishing community North East Brazil [9]. The study revealed that occupants of households with infested pets and domestic animals had tungiasis that correlated to infestation in the animals [9]. The fleas take blood meal from the warm blooded organism and sometimes female *T. penetrans* cause tungiasis in them [7]. Therefore to combat tungiasis in the study areas, separate housing of domestic animals such as goats, chicken, dogs and cats is important. Regularly dusting these animals with insecticides such as sevin dust is crucial to reduce the flea burden.

Poor housing was associated with tungiasis probably because earthen houses are dusty and have cracks on the walls and floors providing good breeding environment for *T. penetrans*. Maintaining high levels of cleanliness in such houses is also quite challenging. This could increase the flea population hence high attack rate. Similar observations were made in a recent study in Murang’a South [11] that demonstrated that the highest chance of infestation exists in individuals living in houses with earthen floors. To decrease transmission of *T. penetrans* among humans, concrete floors should be preferred as opposed to earthen ones. Indeed, in shacks with concrete floors being cleaned every day with water, *T. penetrans* larvae were hardly found [16]. However economically disadvantaged victims, unless supported are not able to improve their housing from the current state. For this reason, we find it important to engage the affected in income generating activities through organized social grouping such as women and youth groups to help combat tungiasis in the study area. This would raise their living standards and afford better housing facilities.

Dusty classrooms including those that are not cemented significantly influence tungiasis in school going children. This is probably because they offer good breeding places. This finding is consistent to a report on tungiasis situation in rural schools in Busia and Teso Districts, Kenya [3] whereby classrooms which are often dusty were identified as points of infestation, which was aggravated by earthen floor. Therefore to reduce tungiasis in such classrooms, daily cleaning of cemented floors with water could be helpful. Sprinkling water on floors that are not cemented, accompanied by sweeping on daily basis would be equally important. Funding of water projects in such schools as well as improving classroom infrastructural standards should utilize Community Development Fund (CDF). This would minimize economic challenges to accessing clean environment.

Regular use of closed footwear could be protective against tungiasis. Generally shoes could prevent infestation and re-infestation of feet by fleas from the ground or from one person to another. This finding is upheld by an earlier study that demonstrated that lack of regular use of proper footwear is an important factor for tungiasis in Erikit, Brazil [8]. Muehlen et al. [15] made similar observation when in his studies on tungiasis concluded that factors such as age, type of housing, level of education, lack of shoes and low socio-economic profile predispose individuals to tungiasis. Shoes however only protect the feet as compared to other parts of the body such as knees or fingers. Furthermore, though shoes are protective to the feet and they do not actually eradicate the fleas. Maintenance of the shoe general hygiene and that of socks to remove and destroys adhered eggs or larvae is quite important. Nevertheless, provision of shoes by County government and NGOs such as Ahadi Kenya Trust would prevent spread of tungiasis to a great extent in pupils in the study areas of Kiharu constituency.

From these observations, there is a strong conviction that improved living standards of the affected communities could reduce tungiasis by a big margin. Intervention by engaging the affected communities in income generating economic activities would help address the economic factors and perhaps tungiasis could be no more.

Kiharu Constituency Strategic Development Plan (2010–2030) [17] acknowledges that tungiasis contribute to school dropout. However it fails to give its prevalence among the other diseases most prevalent in the constituency. A list of most prevalent diseases in the constituency identified in the plan are; malaria 42.4 %, typhoid 14.1 %, flue and cold 12.1 %, Diabetes 8.7 %, pneumonia 6.5 %, STDs & AIDS 5.4 %, tuberculosis 4.2 %, hypertension 2.0 %, stroke 2.0 %, Arthritis 1.7 %, Worm infections 0.6 %, and meningitis 0.3 %. The prevalence of tungiasis is interestingly missing altogether. This is an indication that tungiasis is simply neglected. Heukelbach et al. [18] supports this finding when he pointed out that tungiasis is indeed neglected being considered as just an entomological nuisance. This make it fall sort of attention by researchers and health professionals.

The high prevalence of tungiasis in pupils of 10 to 12 years was probably due to more exposure to associated factors. Most of children at this age are barefooted, playful and may have challenges maintaining cleanliness as well as removing embedded *T. penetrans*. In fact boys of this age spend most of their free time with pets like dogs that could be harboring *T. penetrans* [3]. This perhaps explains their higher prevalence compared to girls, showing preponderance of infestation in the male sex. This finding corresponds to the findings of Community-based studies that have consistently shown preponderance of infestation in the male sex [19, 20]. This trend in prevalence of tungiasis among the age brackets is consistent with observations of another study that demonstrated that a relationship between tungiasis and age brackets exist [8].

This study recommends development and sustenance of public health awareness campaigns to reduce tungiasis. In schools, health clubs or equivalent would be very useful. Strategies against tungiasis aimed at reducing stigma and improving hygiene are important. This study further discourages people from sharing living quarters with domestic animals to reduce chances of infestation. Regular dusting of infested domestic animals with insecticides would reduce the flea burden in such animals. Strategies to engage affected communities in diversified economic activities through organized women and youth groups are highly recommended. This would help address economic factors associated with tungiasis.

## Conclusions

This study concludes that tungiasis is an important health issue in pupils in the selected areas of study in Kiharu constituency that should not be ignored. Intervention strategies addressing the factors associated with the disease should be emphasized to reduce the disease burden. Approaches taken should be holistic involving all community members.
